# Pleiotropic Effects of Polymorphisms in the *BCL11A* Gene on Laboratory Parameters in Sickle Cell Anemia

**DOI:** 10.3390/ijms262110458

**Published:** 2025-10-28

**Authors:** Antonio Mateus Oliveira, Luciana Fiuza, Camylla Figueiredo, Caroline Guarda, Rayra Santiago, Sètondji Yahouédéhou, Suéllen Carvalho, Ana Paula Pacheco, Isa Lyra, Elisângela Vitória Adorno, Cynara Barbosa, Marilda Gonçalves

**Affiliations:** 1 Laboratório de Investigação em Saúde Global e Doenças Negligenciadas, Instituto Gonçalo Moniz, Fundação Oswaldo Cruz—Fiocruz Bahia, Salvador 40296-710, Bahia, Brazil; antoniomateusj@gmail.com (A.M.O.); mfiuza.luciana@gmail.com (L.F.); myllavilas@hotmail.com (C.F.); cguarda4@hotmail.com (C.G.); rayrapsantiago@gmail.com (R.S.); modya006@yahoo.fr (S.Y.); suellen.ufba@gmail.com (S.C.); ana.pacheco@fiocruz.br (A.P.P.); 2 Hospital do Subúrbio, Salvador 40720-460, Bahia, Brazil; isalyra@gmail.com; 3 Laboratório de Pesquisa em Anemias, Departamento de Análises Clínicas e Toxicológicas, Faculdade de Farmácia, Universidade Federal da Bahia, Salvador 40170-110, Bahia, Brazil; elisadorno@ufba.br (E.V.A.); cgbarbosa@ufba.br (C.B.)

**Keywords:** sickle cell anemia, fetal hemoglobin, *BCL11A* polymorphism, HDL, A1AT

## Abstract

Sickle cell anemia (SCA) is characterized by hematological events that lead to vaso-occlusion and the onset of clinical manifestations. Fetal hemoglobin (HbF) has been shown to positively influence the clinical outcomes of individuals with SCA. Genetic polymorphisms are known to modulate clinical phenotypes by increasing HbF levels, with the *BCL11A* gene being an important marker in this regard for future therapies. However, while the *BCL11A* gene plays a role in the regulation of several genes during hematopoiesis, its various effects are not yet fully understood. The study aimed to investigate association between laboratory biomarkers in the presence of rs766432 and rs6732518 polymorphisms in the *BCL11A* gene. Hematological and biochemical markers were analyzed using automated methods, while genetic markers were identified by PCR-RFLP techniques. Elevated HbF levels were significantly associated with the presence of rs766432 and rs6732518 polymorphisms. High concentrations of HDL were associated with rs766432 polymorphism, and elevated levels of alpha-1antitrypsin were linked to the rs6732518 polymorphism. No correlation was found between HbF and HDL concentrations. The low sample size represents a major constraint, making the results only suggestive. In conclusion, polymorphisms in the *BCL11A* gene are important for variations in HbF levels and may have a pleiotropic effect by influencing laboratory parameters unrelated to HbF levels.

## 1. Introduction

Sickle cell anemia (SCA) is a clinical form within the spectrum of sickle cell disease (SCD), determined by a point mutation in the beta-globin gene (*HBB*) in humans, which results in the synthesis of the hemoglobin S (HbS) variant in homozygosis [1]. The change in the red blood cell (RBC) shape, caused by the polymerization of HbS, leads to endothelial damage and makes cells more prone to intravascular hemolysis due to the increased rigidity of sickle-shaped cells [2,3]. The vaso-occlusive processes, primarily in microcirculation, reduce tissue oxygenation, which can result in tissue damage and the ischemia–reperfusion process [4,5]. The progression of the disease is influenced by various factors, including the presence of other comorbidities, intrinsic genetic factors, and the response to drug treatment [6].

Hydroxyurea (HU) is currently recognized as the primary effective drug for the treatment of SCD, acting by increasing plasma levels of fetal hemoglobin (HbF) through gene regulation via pre- and post-transcriptional mechanisms. HU serves as a disease modulator by destabilizing the polymerization of HbS under hypoxic conditions [7,8,9]. Although HU can improve disease progression by directly increasing HbF levels, it is a cytotoxic drug with limited potency and sustainability. Its effectiveness depends on adequate hematopoietic reserves, which are finite [9,10]. Furthermore, its efficacy is influenced by genetic factors that exhibit heterogeneous behavior across different populations, which explains why some patient groups do not respond to the drug. This variability highlights the need for alternative treatments that further promote the elevation of HbF levels [8].

Other treatments for SCD have been tested, but some have yielded controversial results in clinical trials. However, gene therapy offers a promising approach to treating SCD, even though it remains experimental [10]. In vivo studies involving gene editing with CRISPR-Cas9 and lentiviral vectors have produced promising results, including the correction of the mutation that causes SCD, as well as increased HbF levels through the disruption of the *BCL11A* gene [11,12].

HbF production ceases after birth and is replaced by HbS in patients with SCA. This switching process is primarily regulated by BCL11A, a zinc finger regulatory protein encoded by the *BCL11A* gene, which represses the expression of the *HGB1* and *HBG2* genes, thereby promoting the predominance of β chain synthesis, which forms the adult hemoglobin molecule [13,14,15,16,17,18]. The regulatory protein BCL11A plays a key role in modulating the course of SCD, as it directly regulates HbF synthesis. It is responsible for 20–50% of the common variation in HbF levels in both individuals with SCD and healthy adults, and has been extensively studied in both pharmacological and genetic research [9,11,12,19,20].

The presence of SNPs in the *BCL11A* gene has been widely associated with its role in HbF induction. An in silico study investigated 11,463 SNPs obtained through the Database of Single Nucleotide Polymorphisms (dbSNP) and elucidated polymorphisms in the zinc finger domain as suitable targets to disrupt the functions of BCL11A in hemoglobin switching. Therefore, understanding the behavior of SNPs on the functionality of the BCL11A repressor is of importance for clinical studies in SCA [21,22,23].

The SNPs rs766432 (C>A) and rs6732518 (C>T), both located in an intron region in the *BCL11A* gene, are described in the literature as HbF inductors in several populations, such as Iran, Hong Kong and Thailand. These SNPs have also been investigated in Brazilian population, where they exhibit a notable allele frequency. However, their relationship with hematological, biochemical and clinical markers in SCD remains poorly elucidated [24,25,26,27].

In this scenario, the aim of this study was to associate the occurrence of polymorphisms in the *BCL11A* gene with clinical phenotypes in SCA patients without the use of HU. Considering the need to expand treatments for SCD, our work may contribute to future studies in personalized medicine and in the search for targets for treatments based on gene therapy through the conclusive data generated for the genetic plasticity of polymorphisms in different populations.

## 2. Results

### 2.1. Baseline Characteristics of the Study Population

The baseline characteristics of patients with SCA included in this study are described in Table 1.

### 2.2. Allelic and Genotypic Frequencies of Polymorphisms

Genetic analyses identify polymorphisms frequencies in the studied group, in which the allele frequency for the minor allele A of the rs766432 polymorphism showed a lower frequency than the wild-type allele. However, the major allele T allele of the rs6732518 polymorphism showed a higher occurrence in relation to the wild-type allele. Only rs6732518 frequency is in accord to the Hardy–Weinberg equilibrium (HWE) (Table 2).

### 2.3. Comparison Between Genotypes of Polymorphisms and Hemoglobin Levels

**Figure 1:**
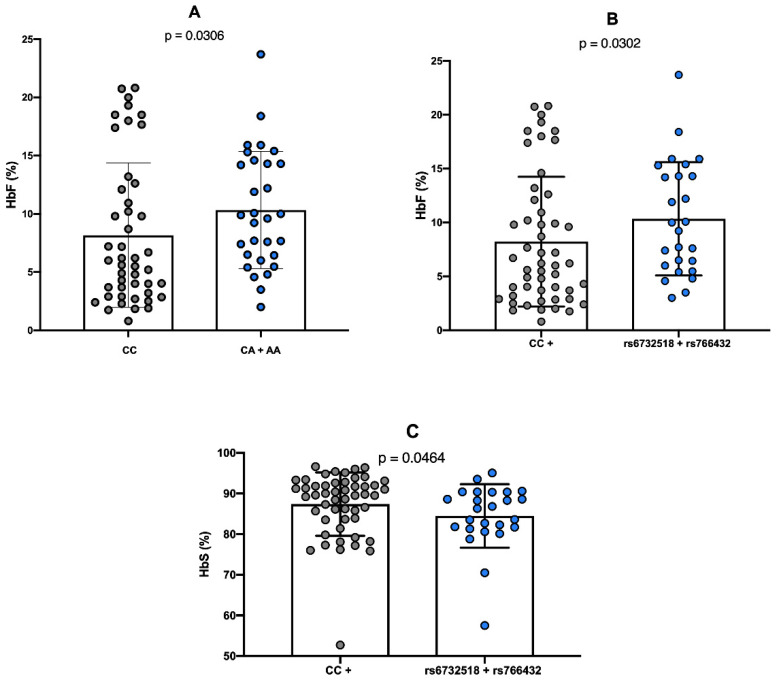
Comparative analysis of HbF levels according to *BCL11A* gene polymorphisms. (**A**) Presence of allele A and elevated HbF levels; (**B**) independent inheritance of minor alleles of rs6732518 and rs766432 polymorphisms and increased HbF levels; (**C**) presence of at least one wild-type genotype of rs6732518 and rs766432 polymorphisms and HbS concentration. The nonparametric Mann–Whitney test was employed to compare the distribution of variables between groups. A *p*-value < 0.05 was considered statistically significant.

Considering the concentrations of HbF and HbS, the presence of the minor allele A allele of the rs766432 polymorphism was significantly associated with elevated HbF levels (*p* = 0.0306), compared to individuals with the wild-type CC genotype (Figure 1A). In contrast, the rs6732518_T polymorphism demonstrated a statistically significant association with HbF levels only when co-inherited with the rs766432_A polymorphism (*p* = 0.0302), relative to individuals carrying the minor alleles of one or neither of the polymorphisms (Figure 1B).

Regarding HbS concentrations, a significant association (*p* = 0.0464) was observed with the co-inheritance of both rs6732518_T and rs766432_A polymorphisms, which was linked to reduced HbS concentration in comparison to individuals harboring only one or neither of the variants (Figure 1C).

### 2.4. Comparison Between Genotypes of Polymorphisms and Laboratory Parameters

**Figure 2:**
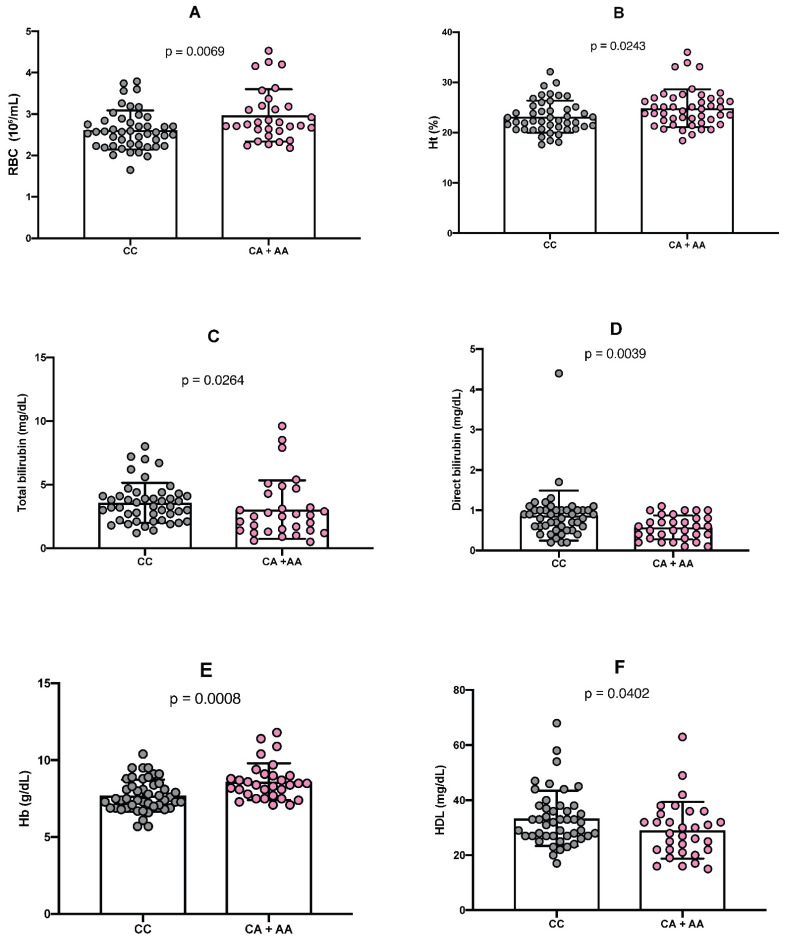
Comparative analysis of laboratory parameters levels according to *BCL11A* gene polymorphisms. (**A**) Differences in red blood cell (RBC) count between major and minor genotypes. (**B**) Comparison of hematocrit (Ht) levels between major and minor genotypes. (**C**) Comparison of total bilirubin levels between wild-type and minor genotypes. (**D**) Comparison of direct bilirubin levels between wild-type and minor genotypes. (**E**) Comparison of hemoglobin (Hb) levels between major and minor genotypes. (**F**) Comparison of LDL cholesterol levels between wild-type and minor genotypes. The nonparametric Mann–Whitney U test was applied to compare the distributions of variables between genotype groups. A *p*-value < 0.05 was considered statistically significant.

Analysis of hematological parameters in individuals carrying the minor allele (A) of the rs766432 polymorphism revealed statistically significant positive differences in the red blood cell count (RBC) (*p* = 0.0069), hematocrit (Ht) (*p* = 0.0243), and hemoglobin (Hb) concentration (*p* = 0.0008). Conversely, significant negative differences were observed for plasma levels of total bilirubin (*p* = 0.0264), indirect bilirubin (*p* = 0.0039), and high-density lipoprotein (HDL) cholesterol (*p* = 0.0402) in the presence of the minor allele (A) of the rs766432 polymorphism (Figure 2).

**Figure 3:**
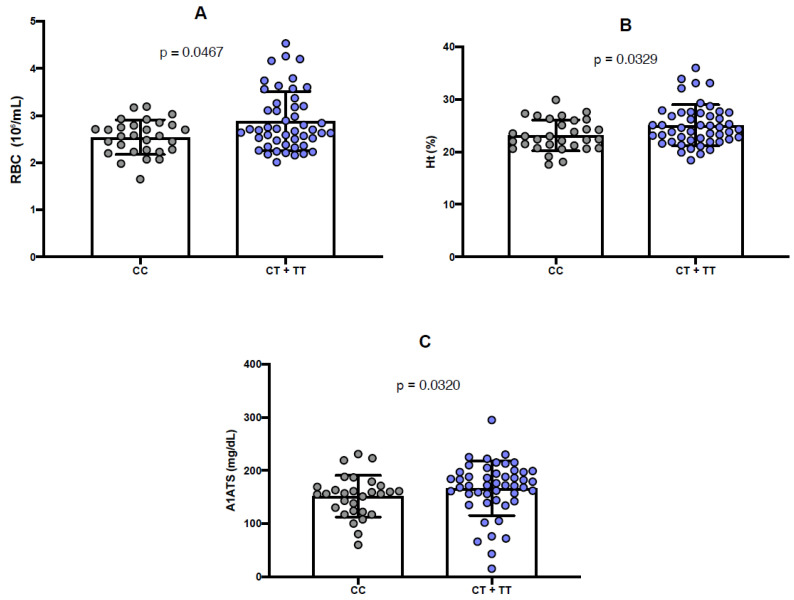
Quantitative analysis of laboratory parameters according to rs6732518 polymorphism genotypes. (**A**) Comparison of red blood cell (RBC) count between major and minor genotypes. (**B**) Comparison of hematocrit (Ht) levels between wild-type and minor genotypes. (**C**) Comparison of alpha-1 antitrypsin (A1AT) levels between major and minor genotypes. The nonparametric Mann–Whitney U test was used to compare variable distribution between genotype groups. A *p*-value < 0.05 was considered statistically significant.

Furthermore, the presence of the minor allele (T) of the rs6732518 polymorphism was significantly linked to increased RBC count (*p* = 0.0467) and Ht values (*p* = 0.0329), compared to the wild-type genotype (Figure 3A,B). Additionally, the T allele was significantly linked to higher levels of alpha-1 antitrypsin (A1AT) (*p* = 0.0320) (Figure 3C).

**Figure 4:**
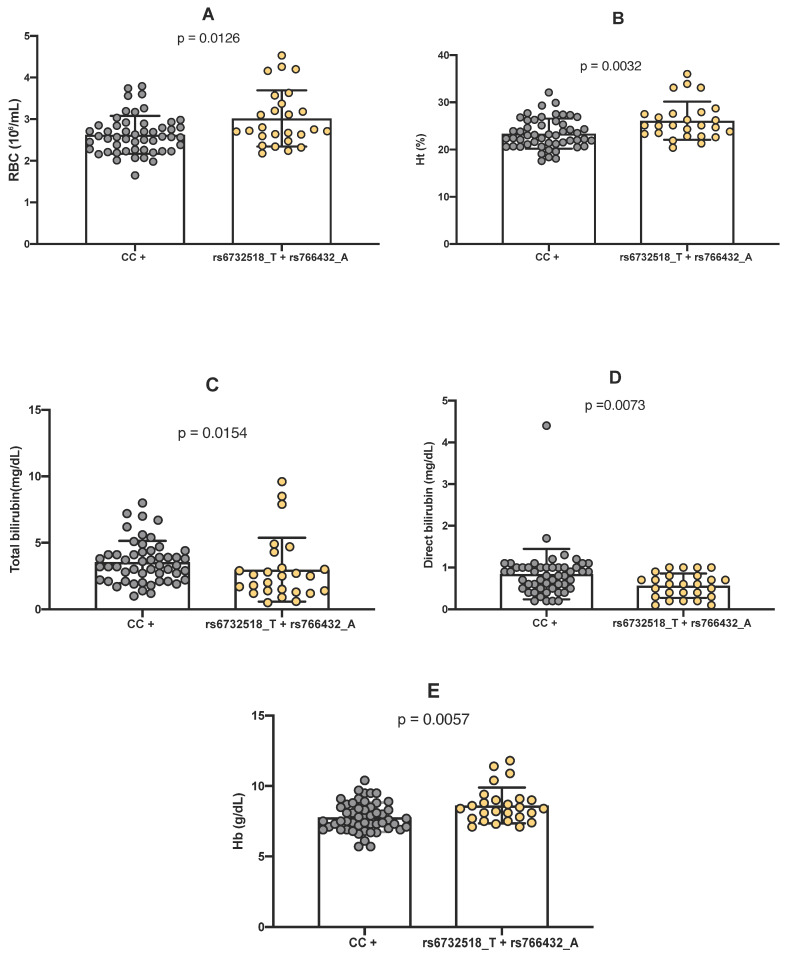
The impact of simultaneous genotypes at rs6732518_T and rs766432_A polymorphisms on laboratory parameters was analyzed. (**A**) Comparison of red blood cell (RBC) count between individuals with and without the simultaneous presence of the minor alleles. (**B**) Comparison of hematocrit (Ht) levels between individuals carrying and not carrying the minor alleles. (**C**) Comparison of total bilirubin levels between individuals presenting at least one homozygous genotype (CC) for the major allele of either polymorphism and those carrying the minor alleles. (**D**) Comparison of direct bilirubin levels between individuals presenting at least one homozygous genotype (CC) for the major allele of the polymorphisms and those carrying the minor alleles. (**E**) Comparison of hemoglobin (Hb) levels between individuals with and without the minor alleles. The nonparametric Mann–Whitney U test was used to compare variable distributions between groups. A *p*-value < 0.05 was considered statistically significant. The symbols (+) denote the presence of any alternative genotype for the respective polymorphism.

Evaluation of the co-inheritance of the minor alleles A (rs766432) and T (rs6732518) in SCD individuals revealed that it was significantly linked to increased RBC count (*p* = 0.0126), Ht (*p* = 0.0032), and Hb (*p* = 0.0057) concentrations (Figure 4A,B,E). Conversely, significantly lower levels of total bilirubin (*p* = 0.00154) and direct bilirubin (*p* = 0.0073) were found in individuals with the dual inheritance of both polymorphism compared to those without this genetic profile characteristic (Figure 4C,D).

### 2.5. Evaluation of the Relationship Between HbF and HDL Cholesterol Levels

**Figure 5:**
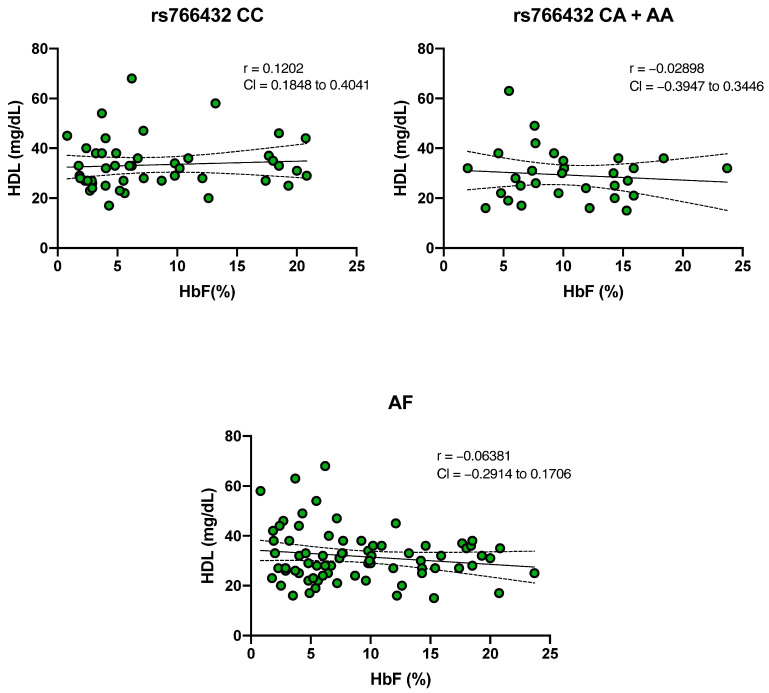
Lack of correlation test was employed to assess the relationship between HDL Cholesterol and fetal hemoglobin (HbF) levels in individuals with sickle cell anemia. The nonparametric Spearman correlation test was employed to assess the relationship between high-density lipoprotein (HDL) cholesterol and fetal hemoglobin (HbF) levels across different groups. The correlation coefficient is represented by r, and CI indicates the confidence interval.

To explore potential associations between the independent variables HbF and HDL cholesterol among SCA individuals, correlation analyses were conducted. No significant correlation was identified with HbF and HDL cholesterol (r = 0.03705), and with the CC wild-type genotype (r = 0.1202), or the minor genotypes CA and AA (r = 0.03705) of the rs766432 polymorphism (Figure 5).

## 3. Discussion

The evaluation of HWE in the present study should be interpreted with caution. In monogenic disorders such as SCA, patient recruitment does not represent a random sample of the general population, and control groups are typically composed of other affected individuals. Therefore, deviations from HWE in this context do not necessarily indicate population stratification, but rather reflect the intrinsic sampling characteristics of rare genetic diseases [28,29]. Fetal hemoglobin (HbF) is a key modulator of clinical severity of SCD, and its regulation through genetic markers has been increasingly elucidated in the scientific literature [9,30,31]. Among these regulators, the *BCL11A* has emerged as a critical gene, identified through by genome-wide association studies (GWAS) as a modulator of HbF levels. It exerts its regulator effect via transcriptional control and epigenetic mechanisms involving chromatin remodeling, positioning it as a major prognostic biomarker for HbF levels [6]. Consequently, recent therapeutic strategies targeting HbF have focused on understanding the molecular mechanisms involving *BCL11A*, including the impact of both synonymous and non-synonymous mutations within its locus. Moreover, recent studies have proposed a pleiotropic role for *BCL11A*, accounting for the phenotypic variability observed in individuals with SCD. This is particularly relevant in cases where clinical, hematological and biochemical phenotypes exhibit discordant biomarker levels [21].

The findings of the present study support the previously reported role of the rs6732518 and rs766432 polymorphisms in the positive regulation of HbF concentration across various populations [24,25,26,27]. Additionally, these results underscore the clinical importance of maintaining elevated HbF levels, either through pharmacologic induction or genetic modulation, to prevent neurological sequelae in SCD, such as stroke in pediatric patients [32]. However, a novel observation emerged: the rs6732518 polymorphism was significantly associated with elevated HbF levels only when co-inherited with the rs766432_A polymorphism. This suggests a potential interaction effect and highlights the need for in vivo studies to elucidate the independent contribution of rs6732518 to HbF regulation. The observed co-inheritance does not necessarily imply synergistic or additive effects, as the combined presence of both polymorphisms does not translate into a straightforward cumulative impact on HbF levels [33]. This effect may be attributable to only one of the SNPs, and with the current data, it was not possible to statistically demonstrate the independence of their effects.

The process of hemoglobin switching, which involves the transition from γ-globin to β-globin chain production, is mediated by *BCL11A* through its binding to the promoters of the *HBG1* and *HBG2* genes. This interaction facilitates the repositioning of the locus control region (LCR) to the *HBB* gene locus, thereby promoting β-globin expression. Given this mechanism, it is expected that polymorphisms impairing *BCL11A*’s repressive function would be associated with reduced levels of sickle hemoglobin (HbS). This study confirmed such an association, but only in individuals with co-inheritance of both rs6732518_T and rs766432_A polymorphisms [16].

Furthermore, the presence of rs766432 polymorphism was associated with favorable hematological phenotypes in individuals with SCD. Specifically, the CA and AA genotypes were linked to higher concentrations of hemoglobin, hematocrit, and RBC count. Hematocrit reflects the proportion of blood composed of former elements, while RBC count provides the cellular concentration per microliter of blood. Elevated values of these parameters in the presence of the rs766432 variant suggest an attenuation of the anemic profile typically observed in SCD, potentially indicating a protective effect conferred by this variant [34,35].

These findings are consistent with previous studies that have reported a beneficial association between the rs766432 polymorphism and laboratory markers in SCD, reinforcing its role as a polymorphism presence and improved hematological indices does not necessarily suggest a distinct role for *BCL11A*. Rather, it likely reflects the indirect effect of elevated HbF levels on clinical and laboratory parameters. Increased HbF concentrations are known to ameliorate disease severity and improve RBC function, thereby enhancing overall hematological profiles in SCD patients [36,37].

In this study, the rs766432 polymorphism was also associated with the altered levels of HDL cholesterol. Specifically, individuals with the CA or AA genotypes exhibited significantly lower HDL concentration. High-density lipoprotein cholesterol is well known for its inverse relationship with cardiovascular risk. Beyond its role in lipid transport, HDL exerts anti-inflammatory and anti-atherogenic effects by facilitating cholesterol efflux, suppressing hematopoietic stem and progenitor cell proliferation, modulating inflammatory responses, and inhibiting inflammasome activation in macrophages [38]. It is also plausible that the HDL alterations we observed may be linked to metabolic derangements in a subset of SCD patients. Indeed, studies have reported that a proportion of SCD individuals fulfill criteria for metabolic syndrome, in which low HDL is a key component [39,40]. Therefore, the observed association with favorable hematological parameters initially appears contradictory.

From a border perspective, HDL exhibits pleiotropic functions during the acute phase inflammatory response. These include neutralization of bacterial endotoxins, promoting of corticosteroid release, inhibition of platelet aggregation, protection of endothelial cell from apoptosis, downregulation of monocytes mediated inflammation, and suppression of endothelial adhesion molecule expression [41]. Prior studies investigating HDL in the context of SCD have reported that hemolytic stress is associated with a significant decline in plasma lipid levels, including HDL cholesterol, which is known to possess both anti-inflammatory and antioxidative properties [42,43,44]. Nevertheless, it is also possible that the observed association with HDL arises from another variant in linkage disequilibrium, instead of representing a direct influence of rs766432_A.

Although the association between the rs766432_A and HDL levels has not been extensively described in the literature, the present findings suggest a pleiotropic effect of this polymorphism in the *BCL11A* gene. Notably, while *BCL11A* is known to induce HbF production thereby improving hematological status and reducing the hemolytic burden, the reduced HDL levels observed in this study conflict with the expected lipid profile in non-hemolytic individuals [45]. The absence of a significant correlation between HbF and HDL levels in this cohort further supports the notion that these two parameters are independently modulated, despite being linked to the same genetic variant. However, although no significant correlation was observed, pleiotropic effects of HbF on blood traits have been previously described in SCD raising the possibility that HbF could influence HDL levels through mechanisms that have not yet been elucidated [46].

Additionally, bilirubin, a key biochemical marker of hemolysis in SCD [21] was found to be significantly decreased in both its total and direct fractions among individuals carrying the rs766432 minor allele. This finding aligns with the proposed protective role of the polymorphisms, corroborating previous study that associated HbF-inducing polymorphisms in *BCLL1A* with improved hematological and biochemical profiles [21].

In parallel, the T allele of the rs6732518 polymorphism also demonstrated associations indicative of a favorable clinical phenotype. Specifically, individuals with the CT or TT genotypes exhibited higher levels of the anti-inflammatory enzyme alpha-1 antitrypsin (A1AT). A1AT, a well-characterized serine protease inhibitor, is abundant in plasma and serves not only as a protease inhibitor but also as a regulator of inflammation and immune responses [47,48,49,50]. Interestingly, elevated A1AT levels have previously been associated with more severe inflammatory phenotypes in SCD [51]. This observation contrasts with our findings, wherein increased A1AT levels were associated with the rs6732518 minor allele, which is generally considered a favorable prognostic marker due to its link with elevated HbF levels [24,25]. These findings suggest that the rs6732518_T polymorphism may exert pleiotropic effects in SCD phenotypes.

Although rs6732518 was not independently associated with total or direct bilirubin levels or RBC count, its co-inheritance with rs766432_A was associated with lower bilirubin concentrations and higher RBC counts. These results imply that the clinical impact of rs6732518_T may be enhanced when inherited alongside other intronic polymorphisms in *BCL11A*, particularly rs766432_A. This observation underscored the need for additional studies to clarify the combinatorial effects of these polymorphisms on hematological and biochemical markers in SCD.

Taken together, these findings have significant implications for future clinical research aimed to understanding the genetic modulation of disease severity in SCD. They also contribute to the characterization of *BCL11A*’s behavior in diverse contexts—a critical objective given that this gene is currently a target in gene therapy strategies for hemoglobinopathies such as SCD. However, it is important to acknowledge the limitations of the present study. The extremely low sample size represents a major constraint and prevents definitive conclusions, making the results only suggestive. In addition, as the conclusions are based on in vitro and in silico analyses, further in vivo studies and functional assays are needed to validate the biological relevance and mechanistic underpinnings of these associations.

## 4. Materials and Methods

### 4.1. Patient Recruitment

A cross-sectional study was conducted to evaluate exposures and outcomes simultaneously. The study was approved by the Human Research Ethics Committee of the Gonçalo Moniz Institute (IGM-FIOCRUZ), under approval number CEP: 29239220.8.3002.8035. Participants with SCA were recruited from the Hematology and Hemotherapy Foundation of the State of Bahia (HEMOBA), where they receive regular follow-up care. This study adhered to the principles of Good Clinical Practice (GCP), the Declaration of Helsinki and its subsequent revisions, as well as Resolution 466/12 and related guidelines of Brazilian National Health Council. All participants provided written informed consent through the Free and Informed Consent Form (FICF) and completed a standardized health questionnaire prior to enrollment.

A total of 77 individuals with SCA were included in this study. Eligibility criteria comprised individuals of any age and sexes with a confirmed HbSS genotype (SCA profile), who had not received hydroxyurea (HU) treatment and were not undergoing chronic blood transfusion therapy. Exclusion criteria included prior or current treatment with HU, cases in which genotypes for the investigated polymorphisms could not be determined, and lack of hemoglobin profile confirmation by high-performance liquid chromatography (HPLC).

### 4.2. Sample Collection

Venous blood samples (5 mL) were collected in EDTA (sodium ethylenediaminetetraacetic acid) tubes (1.5 mg/mL) for hematological, biochemical, hemoglobin profile and genetic analysis.

### 4.3. Hematological and Biochemical Analyses

The hemoglobin profile was assessed using high performance liquid chromatography (HPLC) using the ion exchange principle, performed on a VARIANT^TM^ system (Bio-Rad, Hercules, CA, USA). Hematological parameters and red cell indices were measured using the ABX Pentra 80 automated analyzer (HORIBA Diagnostics, Montpellier, France). Morphological evaluation of red blood cells (RBC) was conducted on Wright-stained peripheral blood smears under optical microscopy. Biochemical analyses, including measurements of total bilirubin and fractions, were performed using an A25 automated analyzer (BioSystems AS, Barcelona, Spain).

### 4.4. Genetic Analyses

Genomic DNA was extracted from 200 µL of peripheral blood using the FlexiGene DNA kit (Qiagen, Germantown, MD, USA) according to the manufacturer’s instructions. Genotyping of *BCL11A* polymorphisms and SCD-associated haplotypes were carried out using polymerase chain reaction followed by restriction fragment length polymorphism analysis (PCR-RFLP) techniques. Primers were designed using the NCBI Primer-BLAST tool (version 2.14.0), and specific primers were synthesized for the amplification of *BCL11A* polymorphisms (Table S1). The sequences were as follows: 5′-TGGGGGTTCAGTGGTTAGAAGG-3′ (forward) and 5′-CCAAATGCTCTGCTTATGGTGG-3′ (reverse) for rs766432. For rs6732518, the following primers were used: 5′-TGGGTGACCCTCTGACTCCT-3′ (forward) and 5′-GCTTTAACGCACTACACCCCAC-3′ (reverse). DNA was stored at −20 °C until analysis.

### 4.5. Statistical Analyses

The distribution of quantitative variables was assessed using the Shapiro–Wilk and D’Agostino tests. Depending on the normality of each variable, either parametric tests (Independent t-test or non-parametric tests (Mann–Whitney U test) were used to compare two groups. Nonparametric Spearman correlation test was applied to evaluate the association between two continuous variables. All statistical analyses were performed using GraphPad Prism version 9.0. Hardy–Weinberg equilibrium (HWE) was evaluated for each polymorphism using the online tool from Tufts University (USA). A *p*-value < 0.05 was considered statistically significant for deviations from HWE.

## 5. Conclusions

This study reinforces the regulatory role of BCL11A in modulating fetal hemoglobin (HbF) levels, a critical factor in the clinical expression of SCD. In addition to this primary role, we identified associations between polymorphisms in the *BCL11A* gene and several laboratory parameters.

Notably, the rs766432 polymorphism showed characteristics of pleiotropy, as it was associated not only with increased HbF levels but also with reduced HDL concentrations. Similarly, the rs6732518 polymorphism was associated with elevated alpha-1 antitrypsin (A1AT) levels. While low HDL is often linked to inflammation, elevated A1AT may represent a compensatory anti-inflammatory response, highlighting the complex and possibly pleiotropic effects of these polymorphisms.

These findings suggest that the *BCL11A* gene influences a broader spectrum of clinical and biochemical features in SCD than previously understood. However, additional studies are warranted to replicate these associations in other populations and to elucidate the underlying molecular mechanisms.

## Figures and Tables

**Table 1:** Baseline characteristics of patients with SCA.

	N	Mean	SD
Gender	77		
Female	33		
Male	44		
Age	77	13.98	9.932
Hematological parameters			
Hemoglobin S, %	77	86.43	7.870
Fetal hemoglobin, %	76	9.019	5.828
Hemoglobin, g/dL	77	8.071	1.186
Hematocrit, %	77	24.29	3.697
RBC, 10^6^/mL	77	5.418	23.28
Biochemical parameters			
Total bilirubin, mg/dL	77	3.360	1.906
Direct bilirubin, mg/dL	77	0.7496	0.5337
Lipid metabolism			
HDL, mg/dL	77	31.65	10.32

HDL: High-density lipoprotein cholesterol; RBC: Red blood cells; SD: Standard deviation.

**Table 2:** Allelic frequencies of rs6732518_T and rs766432_A polymorphisms.

Polymorphism	Genotype	Freq. (%)	Allele	Freq.	EHW	X^2^
rs766432 C>A	CC	59.74	C	0.77	no	0.0002
	CA	35.06	A	0.23		
	AA	5.19				
rs6732518 C>T	CC	37.66	C	0.38	yes	0.20032
	CT	49.35	T	0.62		
	TT	12.98				

EHW: Hardy–Weinberg equilibrium; Freq.: Frequency.

## Data Availability

The original contributions presented in this study are included in the article/Supplementary Materials. Further inquiries can be directed to the corresponding author.

## Supplementary Materials

The following supporting information can be downloaded at: https://www.mdpi.com/article/10.3390/ijms262110458/s1.
