# Supplementary material for: Pleiotropic Effects of Polymorphisms in the BCL11A Gene on Laboratory Parameters in Sickle Cell Anemia
Source: Int J Mol Sci. 2025 Oct 28;26(21):10458. doi: 10.3390/ijms262110458 (PMC12609289; doi:10.3390/ijms262110458)
Supplement: Supplementary file 1 [file ijms-26-10458-s001.zip › ijms-3899960-supplementary.pdf]

Supplementary Table S1. Primers sequences for amplification by PCR.

Polymorphism	Forward sequence	Reverse sequence
rs766432 C>A	TgggggTTCAgTggTTAgAAgg	CCAAATgCTCTgCTTATggTgg
rs6732518 C>T	TgggTgACCCTCTgACTCCT	gCTTTAACgCACTACACCCCAC
